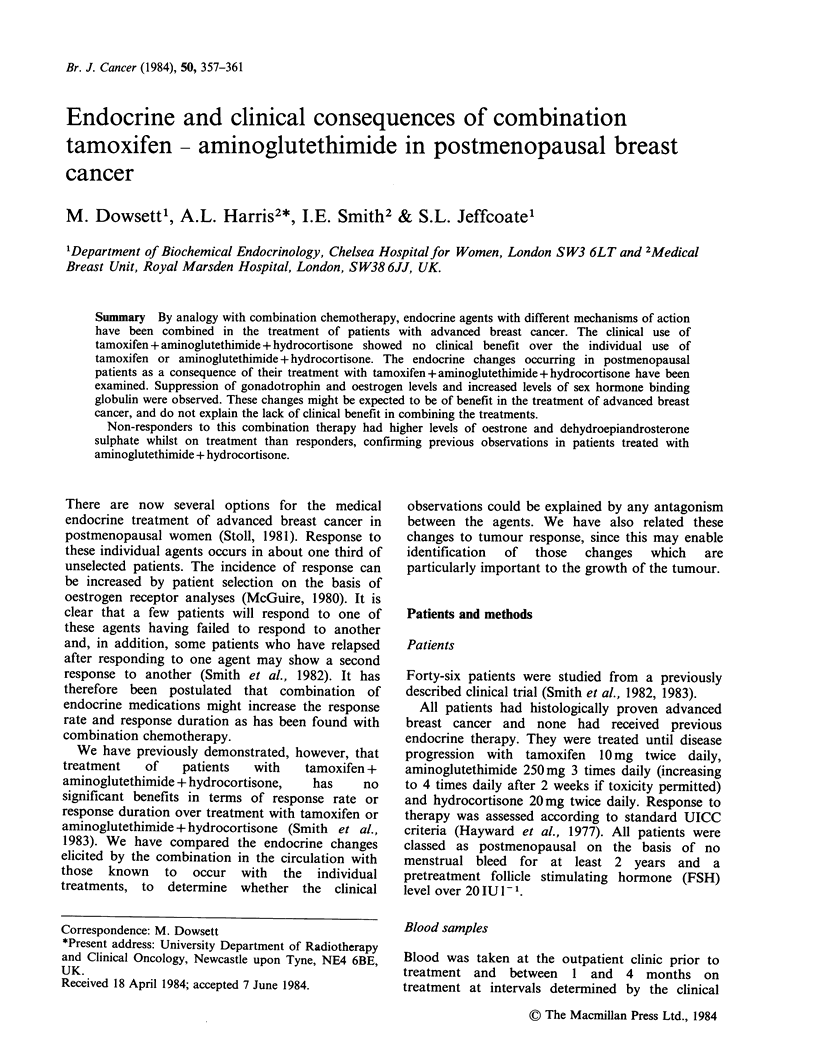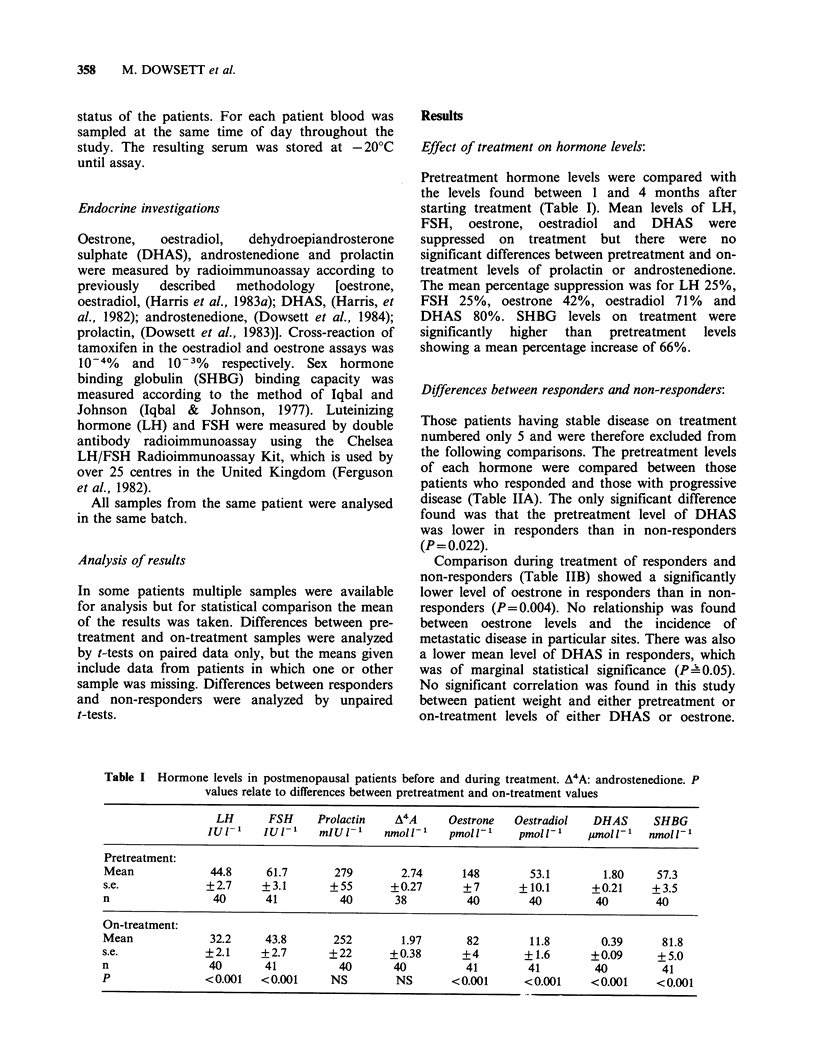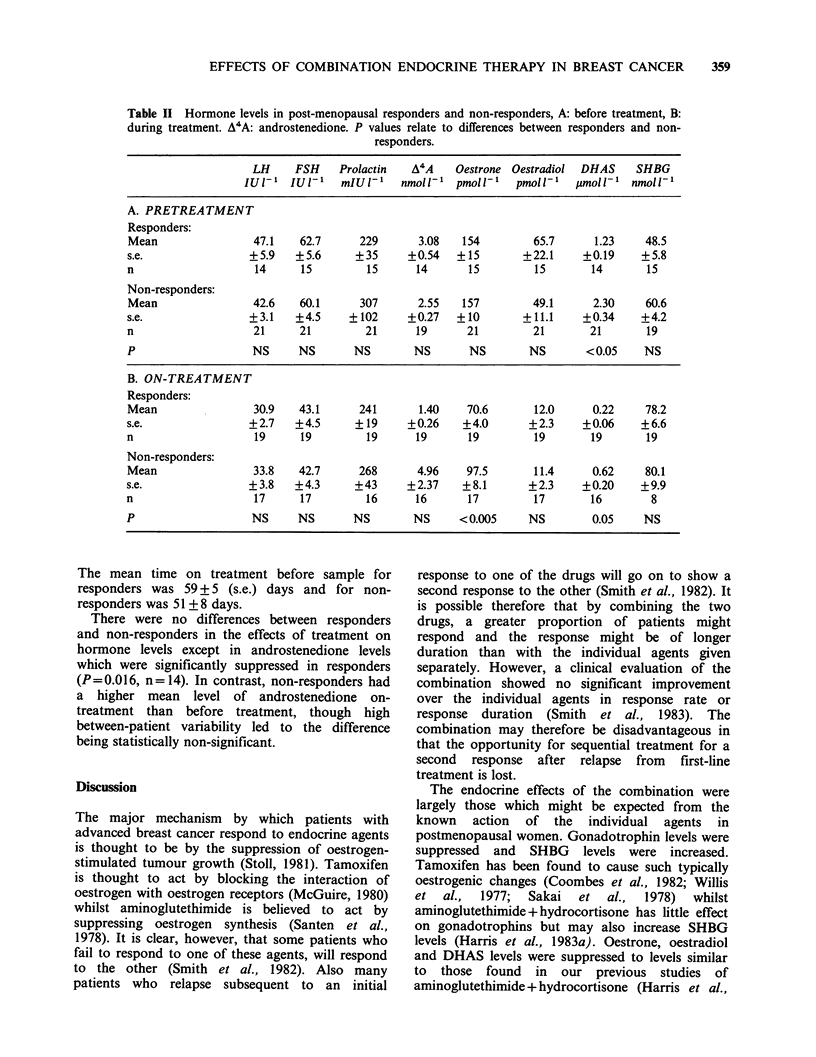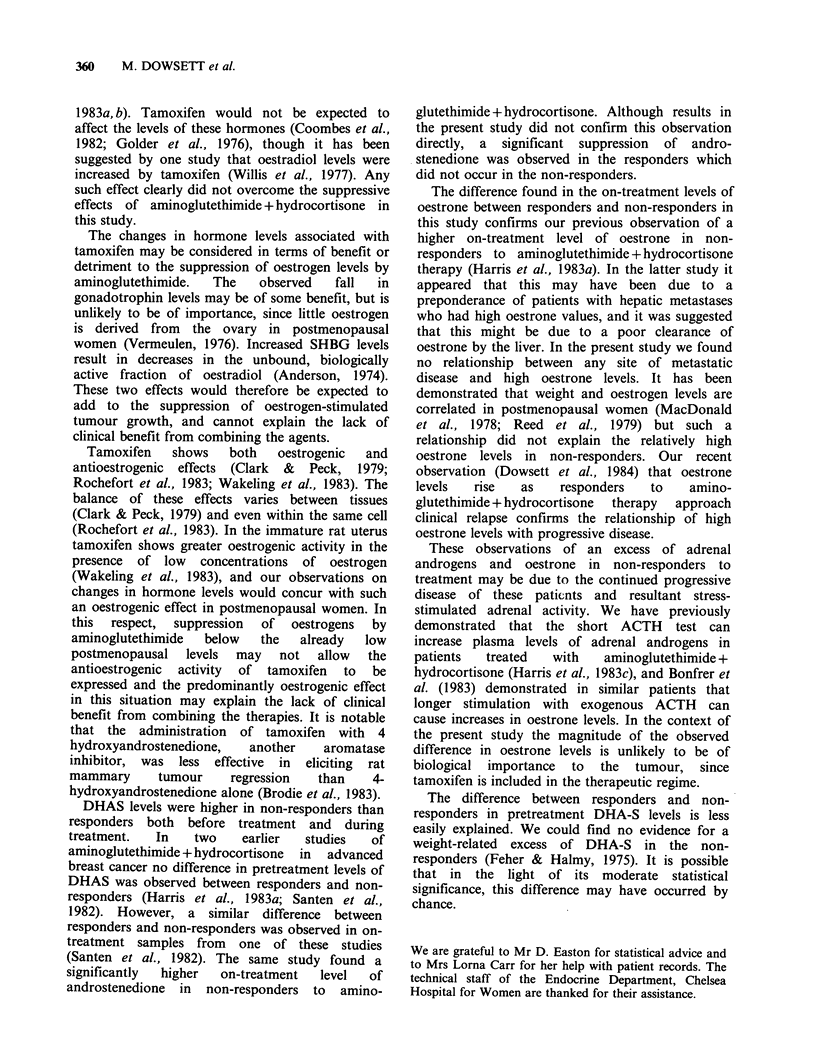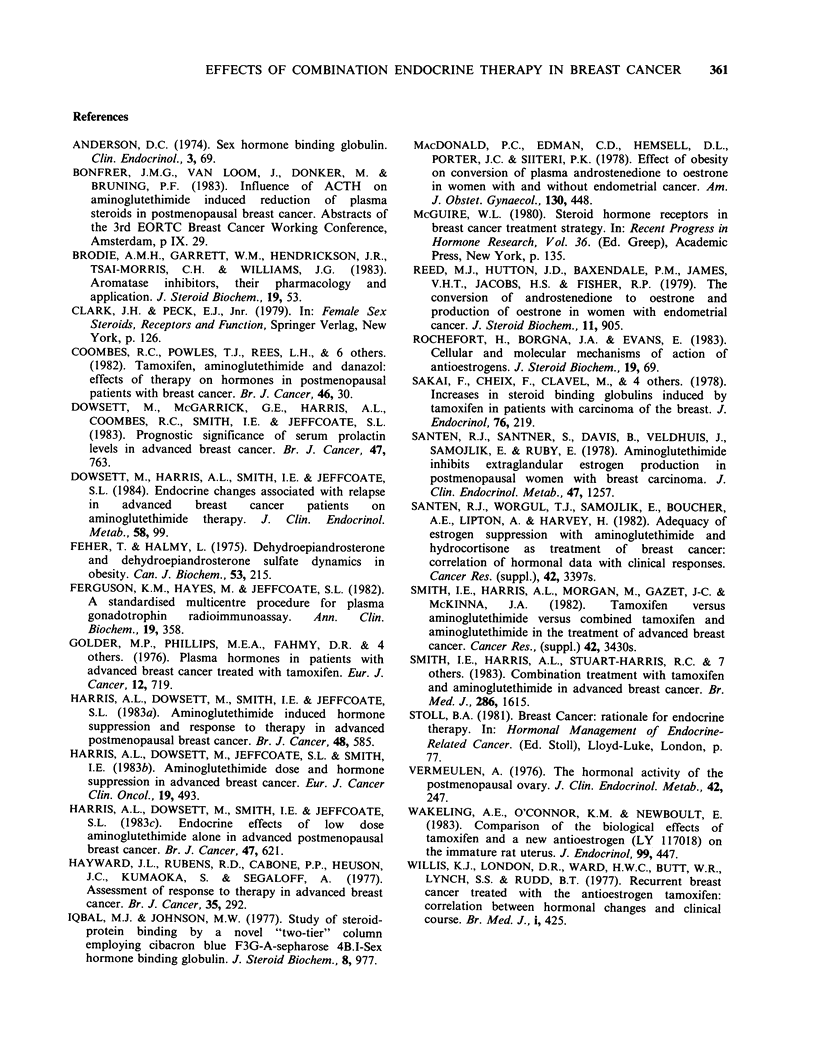# Endocrine and clinical consequences of combination tamoxifen-aminoglutethimide in postmenopausal breast cancer.

**DOI:** 10.1038/bjc.1984.183

**Published:** 1984-09

**Authors:** M. Dowsett, A. L. Harris, I. E. Smith, S. L. Jeffcoate

## Abstract

By analogy with combination chemotherapy, endocrine agents with different mechanisms of action have been combined in the treatment of patients with advanced breast cancer. The clinical use of tamoxifen+aminoglutethimide+hydrocortisone showed no clinical benefit over the individual use of tamoxifen or aminoglutethimide+hydrocortisone. The endocrine changes occurring in postmenopausal patients as a consequence of their treatment with tamoxifen+aminoglutethimide+hydrocortisone have been examined. Suppression of gonadotrophin and oestrogen levels and increased levels of sex hormone binding globulin were observed. These changes might be expected to be of benefit in the treatment of advanced breast cancer, and do not explain the lack of clinical benefit in combining the treatments. Non-responders to this combination therapy had higher levels of oestrone and dehydroepiandrosterone sulphate whilst on treatment than responders, confirming previous observations in patients treated with aminoglutethimide+hydrocortisone.


Br. J. Cancer (1984), 50, 357–361

### Endocrine and clinical consequences of combination tamoxifen – aminoglutethimide in postmenopausal breast cancer

M. Dowsett[1], A.L. Harris[2]*, I.E. Smith[2] & S.L. Jeffcoate[1]

[1]*Department of Biochemical Endocrinology, Chelsea Hospital for Women, London SW3 6LT and* [2]*Medical Breast Unit, Royal Marsden Hospital, London, SW38 6JJ, UK.*

**Summary** By analogy with combination chemotherapy, endocrine agents with different mechanisms of action have been combined in the treatment of patients with advanced breast cancer. The clinical use of tamoxifen + aminoglutethimide + hydrocortisone showed no clinical benefit over the individual use of tamoxifen or aminoglutethimide + hydrocortisone. The endocrine changes occurring in postmenopausal patients as a consequence of their treatment with tamoxifen + aminoglutethimide + hydrocortisone have been examined. Suppression of gonadotrophin and oestrogen levels and increased levels of sex hormone binding globulin were observed. These changes might be expected to be of benefit in the treatment of advanced breast cancer, and do not explain the lack of clinical benefit in combining the treatments.

Non-responders to this combination therapy had higher levels of oestrone and dehydroepiandrosterone sulphate whilst on treatment than responders, confirming previous observations in patients treated with aminoglutethimide + hydrocortisone.

There are now several options for the medical endocrine treatment of advanced breast cancer in postmenopausal women (Stoll, 1981). Response to these individual agents occurs in about one third of unselected patients. The incidence of response can be increased by patient selection on the basis of oestrogen receptor analyses (McGuire, 1980). It is clear that a few patients will respond to one of these agents having failed to respond to another and, in addition, some patients who have relapsed after responding to one agent may show a second response to another (Smith *et al.*, 1982). It has therefore been postulated that combination of endocrine medications might increase the response rate and response duration as has been found with combination chemotherapy.

We have previously demonstrated, however, that treatment of patients with tamoxifen + aminoglutethimide + hydrocortisone, has no significant benefits in terms of response rate or response duration over treatment with tamoxifen or aminoglutethimide + hydrocortisone (Smith *et al.*, 1983). We have compared the endocrine changes elicited by the combination in the circulation with those known to occur with the individual treatments, to determine whether the clinical observations could be explained by any antagonism between the agents. We have also related these changes to tumour response, since this may enable identification of those changes which are particularly important to the growth of the tumour.

Correspondence: M. Dowsett
*Present address: University Department of Radiotherapy and Clinical Oncology, Newcastle upon Tyne, NE4 6BE, UK.
Received 18 April 1984; accepted 7 June 1984.

### Patients and methods

### *Patients*

Forty-six patients were studied from a previously described clinical trial (Smith *et al.*, 1982, 1983).

All patients had histologically proven advanced breast cancer and none had received previous endocrine therapy. They were treated until disease progression with tamoxifen 10 mg twice daily, aminoglutethimide 250 mg 3 times daily (increasing to 4 times daily after 2 weeks if toxicity permitted) and hydrocortisone 20 mg twice daily. Response to therapy was assessed according to standard UICC criteria (Hayward *et al.*, 1977). All patients were classed as postmenopausal on the basis of no menstrual bleed for at least 2 years and a pretreatment follicle stimulating hormone (FSH) level over 20 IU $l^{-1}$.

### *Blood samples*

Blood was taken at the outpatient clinic prior to treatment and between 1 and 4 months on treatment at intervals determined by the clinical

© The Macmillan Press Ltd., 1984

status of the patients. For each patient blood was sampled at the same time of day throughout the study. The resulting serum was stored at −20°C until assay.

*Endocrine investigations*

Oestrone, oestradiol, dehydroepiandrosterone sulphate (DHAS), androstenedione and prolactin were measured by radioimmunoassay according to previously described methodology [oestrone, oestradiol, (Harris *et al.*, 1983*a*); DHAS, (Harris, *et al.*, 1982); androstenedione, (Dowsett *et al.*, 1984); prolactin, (Dowsett *et al.*, 1983)]. Cross-reaction of tamoxifen in the oestradiol and oestrone assays was $10^{-4}$% and $10^{-3}$% respectively. Sex hormone binding globulin (SHBG) binding capacity was measured according to the method of Iqbal and Johnson (Iqbal & Johnson, 1977). Luteinizing hormone (LH) and FSH were measured by double antibody radioimmunoassay using the Chelsea LH/FSH Radioimmunoassay Kit, which is used by over 25 centres in the United Kingdom (Ferguson *et al.*, 1982).

All samples from the same patient were analysed in the same batch.

*Analysis of results*

In some patients multiple samples were available for analysis but for statistical comparison the mean of the results was taken. Differences between pre-treatment and on-treatment samples were analyzed by *t*-tests on paired data only, but the means given include data from patients in which one or other sample was missing. Differences between responders and non-responders were analyzed by unpaired *t*-tests.

**Results**

*Effect of treatment on hormone levels*:

Pretreatment hormone levels were compared with the levels found between 1 and 4 months after starting treatment (Table I). Mean levels of LH, FSH, oestrone, oestradiol and DHAS were suppressed on treatment but there were no significant differences between pretreatment and on-treatment levels of prolactin or androstenedione. The mean percentage suppression was for LH 25%, FSH 25%, oestrone 42%, oestradiol 71% and DHAS 80%. SHBG levels on treatment were significantly higher than pretreatment levels showing a mean percentage increase of 66%.

*Differences between responders and non-responders*:

Those patients having stable disease on treatment numbered only 5 and were therefore excluded from the following comparisons. The pretreatment levels of each hormone were compared between those patients who responded and those with progressive disease (Table IIA). The only significant difference found was that the pretreatment level of DHAS was lower in responders than in non-responders ($P=0.022$).

Comparison during treatment of responders and non-responders (Table IIB) showed a significantly lower level of oestrone in responders than in non-responders ($P=0.004$). No relationship was found between oestrone levels and the incidence of metastatic disease in particular sites. There was also a lower mean level of DHAS in responders, which was of marginal statistical significance ($P \doteq 0.05$). No significant correlation was found in this study between patient weight and either pretreatment or on-treatment levels of either DHAS or oestrone.

**Table I** Hormone levels in postmenopausal patients before and during treatment. $\Delta^4$A: androstenedione. *P* values relate to differences between pretreatment and on-treatment values

| | *LH* $IU\,l^{-1}$ | *FSH* $IU\,l^{-1}$ | *Prolactin* $mIU\,l^{-1}$ | $\Delta^4A$ $nmol\,l^{-1}$ | *Oestrone* $pmol\,l^{-1}$ | *Oestradiol* $pmol\,l^{-1}$ | *DHAS* $\mu mol\,l^{-1}$ | *SHBG* $nmol\,l^{-1}$ |
|---|---|---|---|---|---|---|---|---|
| Pretreatment: | | | | | | | | |
| Mean | 44.8 | 61.7 | 279 | 2.74 | 148 | 53.1 | 1.80 | 57.3 |
| s.e. | ±2.7 | ±3.1 | ±55 | ±0.27 | ±7 | ±10.1 | ±0.21 | ±3.5 |
| n | 40 | 41 | 40 | 38 | 40 | 40 | 40 | 40 |
| On-treatment: | | | | | | | | |
| Mean | 32.2 | 43.8 | 252 | 1.97 | 82 | 11.8 | 0.39 | 81.8 |
| s.e. | ±2.1 | ±2.7 | ±22 | ±0.38 | ±4 | ±1.6 | ±0.09 | ±5.0 |
| n | 40 | 41 | 40 | 40 | 41 | 41 | 40 | 41 |
| *P* | <0.001 | <0.001 | NS | NS | <0.001 | <0.001 | <0.001 | <0.001 |

**Table II** Hormone levels in post-menopausal responders and non-responders, A: before treatment, B: during treatment. $\Delta^4$A: androstenedione. *P* values relate to differences between responders and non-responders.

| | *LH* $IU\,l^{-1}$ | *FSH* $IU\,l^{-1}$ | *Prolactin* $mIU\,l^{-1}$ | $\Delta^4 A$ $nmol\,l^{-1}$ | *Oestrone* $pmol\,l^{-1}$ | *Oestradiol* $pmol\,l^{-1}$ | *DHAS* $\mu mol\,l^{-1}$ | *SHBG* $nmol\,l^{-1}$ |
|---|---|---|---|---|---|---|---|---|
| A. *PRETREATMENT* | | | | | | | | |
| Responders: | | | | | | | | |
| Mean | 47.1 | 62.7 | 229 | 3.08 | 154 | 65.7 | 1.23 | 48.5 |
| s.e. | ±5.9 | ±5.6 | ±35 | ±0.54 | ±15 | ±22.1 | ±0.19 | ±5.8 |
| n | 14 | 15 | 15 | 14 | 15 | 15 | 14 | 15 |
| Non-responders: | | | | | | | | |
| Mean | 42.6 | 60.1 | 307 | 2.55 | 157 | 49.1 | 2.30 | 60.6 |
| s.e. | ±3.1 | ±4.5 | ±102 | ±0.27 | ±10 | ±11.1 | ±0.34 | ±4.2 |
| n | 21 | 21 | 21 | 19 | 21 | 21 | 21 | 19 |
| *P* | NS | NS | NS | NS | NS | NS | <0.05 | NS |
| B. *ON-TREATMENT* | | | | | | | | |
| Responders: | | | | | | | | |
| Mean | 30.9 | 43.1 | 241 | 1.40 | 70.6 | 12.0 | 0.22 | 78.2 |
| s.e. | ±2.7 | ±4.5 | ±19 | ±0.26 | ±4.0 | ±2.3 | ±0.06 | ±6.6 |
| n | 19 | 19 | 19 | 19 | 19 | 19 | 19 | 19 |
| Non-responders: | | | | | | | | |
| Mean | 33.8 | 42.7 | 268 | 4.96 | 97.5 | 11.4 | 0.62 | 80.1 |
| s.e. | ±3.8 | ±4.3 | ±43 | ±2.37 | ±8.1 | ±2.3 | ±0.20 | ±9.9 |
| n | 17 | 17 | 16 | 16 | 17 | 17 | 16 | 8 |
| *P* | NS | NS | NS | NS | <0.005 | NS | 0.05 | NS |

The mean time on treatment before sample for responders was 59±5 (s.e.) days and for non-responders was 51±8 days.

There were no differences between responders and non-responders in the effects of treatment on hormone levels except in androstenedione levels which were significantly suppressed in responders ($P=0.016$, n=14). In contrast, non-responders had a higher mean level of androstenedione on-treatment than before treatment, though high between-patient variability led to the difference being statistically non-significant.

### Discussion

The major mechanism by which patients with advanced breast cancer respond to endocrine agents is thought to be by the suppression of oestrogen-stimulated tumour growth (Stoll, 1981). Tamoxifen is thought to act by blocking the interaction of oestrogen with oestrogen receptors (McGuire, 1980) whilst aminoglutethimide is believed to act by suppressing oestrogen synthesis (Santen *et al.*, 1978). It is clear, however, that some patients who fail to respond to one of these agents, will respond to the other (Smith *et al.*, 1982). Also many patients who relapse subsequent to an initial response to one of the drugs will go on to show a second response to the other (Smith *et al.*, 1982). It is possible therefore that by combining the two drugs, a greater proportion of patients might respond and the response might be of longer duration than with the individual agents given separately. However, a clinical evaluation of the combination showed no significant improvement over the individual agents in response rate or response duration (Smith *et al.*, 1983). The combination may therefore be disadvantageous in that the opportunity for sequential treatment for a second response after relapse from first-line treatment is lost.

The endocrine effects of the combination were largely those which might be expected from the known action of the individual agents in postmenopausal women. Gonadotrophin levels were suppressed and SHBG levels were increased. Tamoxifen has been found to cause such typically oestrogenic changes (Coombes *et al.*, 1982; Willis *et al.*, 1977; Sakai *et al.*, 1978) whilst aminoglutethimide+hydrocortisone has little effect on gonadotrophins but may also increase SHBG levels (Harris *et al.*, 1983*a*). Oestrone, oestradiol and DHAS levels were suppressed to levels similar to those found in our previous studies of aminoglutethimide+hydrocortisone (Harris *et al.*,

1983*a,b*). Tamoxifen would not be expected to affect the levels of these hormones (Coombes *et al.*, 1982; Golder *et al.*, 1976), though it has been suggested by one study that oestradiol levels were increased by tamoxifen (Willis *et al.*, 1977). Any such effect clearly did not overcome the suppressive effects of aminoglutethimide + hydrocortisone in this study.

The changes in hormone levels associated with tamoxifen may be considered in terms of benefit or detriment to the suppression of oestrogen levels by aminoglutethimide. The observed fall in gonadotrophin levels may be of some benefit, but is unlikely to be of importance, since little oestrogen is derived from the ovary in postmenopausal women (Vermeulen, 1976). Increased SHBG levels result in decreases in the unbound, biologically active fraction of oestradiol (Anderson, 1974). These two effects would therefore be expected to add to the suppression of oestrogen-stimulated tumour growth, and cannot explain the lack of clinical benefit from combining the agents.

Tamoxifen shows both oestrogenic and antioestrogenic effects (Clark & Peck, 1979; Rochefort *et al.*, 1983; Wakeling *et al.*, 1983). The balance of these effects varies between tissues (Clark & Peck, 1979) and even within the same cell (Rochefort *et al.*, 1983). In the immature rat uterus tamoxifen shows greater oestrogenic activity in the presence of low concentrations of oestrogen (Wakeling *et al.*, 1983), and our observations on changes in hormone levels would concur with such an oestrogenic effect in postmenopausal women. In this respect, suppression of oestrogens by aminoglutethimide below the already low postmenopausal levels may not allow the antioestrogenic activity of tamoxifen to be expressed and the predominantly oestrogenic effect in this situation may explain the lack of clinical benefit from combining the therapies. It is notable that the administration of tamoxifen with 4 hydroxyandrostenedione, another aromatase inhibitor, was less effective in eliciting rat mammary tumour regression than 4-hydroxyandrostenedione alone (Brodie *et al.*, 1983).

DHAS levels were higher in non-responders than responders both before treatment and during treatment. In two earlier studies of aminoglutethimide + hydrocortisone in advanced breast cancer no difference in pretreatment levels of DHAS was observed between responders and non-responders (Harris *et al.*, 1983*a*; Santen *et al.*, 1982). However, a similar difference between responders and non-responders was observed in on-treatment samples from one of these studies (Santen *et al.*, 1982). The same study found a significantly higher on-treatment level of androstenedione in non-responders to aminoglutethimide + hydrocortisone. Although results in the present study did not confirm this observation directly, a significant suppression of androstenedione was observed in the responders which did not occur in the non-responders.

The difference found in the on-treatment levels of oestrone between responders and non-responders in this study confirms our previous observation of a higher on-treatment level of oestrone in non-responders to aminoglutethimide + hydrocortisone therapy (Harris *et al.*, 1983*a*). In the latter study it appeared that this may have been due to a preponderance of patients with hepatic metastases who had high oestrone values, and it was suggested that this might be due to a poor clearance of oestrone by the liver. In the present study we found no relationship between any site of metastatic disease and high oestrone levels. It has been demonstrated that weight and oestrogen levels are correlated in postmenopausal women (MacDonald *et al.*, 1978; Reed *et al.*, 1979) but such a relationship did not explain the relatively high oestrone levels in non-responders. Our recent observation (Dowsett *et al.*, 1984) that oestrone levels rise as responders to aminoglutethimide + hydrocortisone therapy approach clinical relapse confirms the relationship of high oestrone levels with progressive disease.

These observations of an excess of adrenal androgens and oestrone in non-responders to treatment may be due to the continued progressive disease of these patients and resultant stress-stimulated adrenal activity. We have previously demonstrated that the short ACTH test can increase plasma levels of adrenal androgens in patients treated with aminoglutethimide + hydrocortisone (Harris *et al.*, 1983*c*), and Bonfrer *et al.* (1983) demonstrated in similar patients that longer stimulation with exogenous ACTH can cause increases in oestrone levels. In the context of the present study the magnitude of the observed difference in oestrone levels is unlikely to be of biological importance to the tumour, since tamoxifen is included in the therapeutic regime.

The difference between responders and non-responders in pretreatment DHA-S levels is less easily explained. We could find no evidence for a weight-related excess of DHA-S in the non-responders (Feher & Halmy, 1975). It is possible that in the light of its moderate statistical significance, this difference may have occurred by chance.

We are grateful to Mr D. Easton for statistical advice and to Mrs Lorna Carr for her help with patient records. The technical staff of the Endocrine Department, Chelsea Hospital for Women are thanked for their assistance.

**References**

ANDERSON, D.C. (1974). Sex hormone binding globulin. *Clin. Endocrinol.*, **3**, 69.

BONFRER, J.M.G., VAN LOOM, J., DONKER, M. & BRUNING, P.F. (1983). Influence of ACTH on aminoglutethimide induced reduction of plasma steroids in postmenopausal breast cancer. Abstracts of the 3rd EORTC Breast Cancer Working Conference, Amsterdam, p IX. 29.

BRODIE, A.M.H., GARRETT, W.M., HENDRICKSON, J.R., TSAI-MORRIS, C.H. & WILLIAMS, J.G. (1983). Aromatase inhibitors, their pharmacology and application. *J. Steroid Biochem.*, **19**, 53.

CLARK, J.H. & PECK, E.J., Jnr. (1979). In: *Female Sex Steroids, Receptors and Function*, Springer Verlag, New York, p. 126.

COOMBES, R.C., POWLES, T.J., REES, L.H., & 6 others. (1982). Tamoxifen, aminoglutethimide and danazol: effects of therapy on hormones in postmenopausal patients with breast cancer. *Br. J. Cancer*, **46**, 30.

DOWSETT, M., McGARRICK, G.E., HARRIS, A.L., COOMBES, R.C., SMITH, I.E. & JEFFCOATE, S.L. (1983). Prognostic significance of serum prolactin levels in advanced breast cancer. *Br. J. Cancer*, **47**, 763.

DOWSETT, M., HARRIS, A.L., SMITH, I.E. & JEFFCOATE, S.L. (1984). Endocrine changes associated with relapse in advanced breast cancer patients on aminoglutethimide therapy. *J. Clin. Endocrinol. Metab.*, **58**, 99.

FEHER, T. & HALMY, L. (1975). Dehydroepiandrosterone and dehydroepiandrosterone sulfate dynamics in obesity. *Can. J. Biochem.*, **53**, 215.

FERGUSON, K.M., HAYES, M. & JEFFCOATE, S.L. (1982). A standardised multicentre procedure for plasma gonadotrophin radioimmunoassay. *Ann. Clin. Biochem.*, **19**, 358.

GOLDER, M.P., PHILLIPS, M.E.A., FAHMY, D.R. & 4 others. (1976). Plasma hormones in patients with advanced breast cancer treated with tamoxifen. *Eur. J. Cancer*, **12**, 719.

HARRIS, A.L., DOWSETT, M., SMITH, I.E. & JEFFCOATE, S.L. (1983*a*). Aminoglutethimide induced hormone suppression and response to therapy in advanced postmenopausal breast cancer. *Br. J. Cancer*, **48**, 585.

HARRIS, A.L., DOWSETT, M., JEFFCOATE, S.L. & SMITH, I.E. (1983*b*). Aminoglutethimide dose and hormone suppression in advanced breast cancer. *Eur. J. Cancer Clin. Oncol.*, **19**, 493.

HARRIS, A.L., DOWSETT, M., SMITH, I.E. & JEFFCOATE, S.L. (1983*c*). Endocrine effects of low dose aminoglutethimide alone in advanced postmenopausal breast cancer. *Br. J. Cancer*, **47**, 621.

HAYWARD, J.L., RUBENS, R.D., CABONE, P.P., HEUSON, J.C., KUMAOKA, S. & SEGALOFF, A. (1977). Assessment of response to therapy in advanced breast cancer. *Br. J. Cancer*, **35**, 292.

IQBAL, M.J. & JOHNSON, M.W. (1977). Study of steroid-protein binding by a novel "two-tier" column employing cibacron blue F3G-A-sepharose 4B.I-Sex hormone binding globulin. *J. Steroid Biochem.*, **8**, 977.

MacDONALD, P.C., EDMAN, C.D., HEMSELL, D.L., PORTER, J.C. & SIITERI, P.K. (1978). Effect of obesity on conversion of plasma androstenedione to oestrone in women with and without endometrial cancer. *Am. J. Obstet. Gynaecol.*, **130**, 448.

McGUIRE, W.L. (1980). Steroid hormone receptors in breast cancer treatment strategy. In: *Recent Progress in Hormone Research, Vol. 36*. (Ed. Greep), Academic Press, New York, p. 135.

REED, M.J., HUTTON, J.D., BAXENDALE, P.M., JAMES, V.H.T., JACOBS, H.S. & FISHER, R.P. (1979). The conversion of androstenedione to oestrone and production of oestrone in women with endometrial cancer. *J. Steroid Biochem.*, **11**, 905.

ROCHEFORT, H., BORGNA, J.A. & EVANS, E. (1983). Cellular and molecular mechanisms of action of antioestrogens. *J. Steroid Biochem.*, **19**, 69.

SAKAI, F., CHEIX, F., CLAVEL, M., & 4 others. (1978). Increases in steroid binding globulins induced by tamoxifen in patients with carcinoma of the breast. *J. Endocrinol*, **76**, 219.

SANTEN, R.J., SANTNER, S., DAVIS, B., VELDHUIS, J., SAMOJLIK, E. & RUBY, E. (1978). Aminoglutethimide inhibits extraglandular estrogen production in postmenopausal women with breast carcinoma. *J. Clin. Endocrinol. Metab.*, **47**, 1257.

SANTEN, R.J., WORGUL, T.J., SAMOJLIK, E., BOUCHER, A.E., LIPTON, A. & HARVEY, H. (1982). Adequacy of estrogen suppression with aminoglutethimide and hydrocortisone as treatment of breast cancer: correlation of hormonal data with clinical responses. *Cancer Res.* (suppl.), **42**, 3397s.

SMITH, I.E., HARRIS, A.L., MORGAN, M., GAZET, J-C. & McKINNA, J.A. (1982). Tamoxifen versus aminoglutethimide versus combined tamoxifen and aminoglutethimide in the treatment of advanced breast cancer. *Cancer Res.*, (suppl.) **42**, 3430s.

SMITH, I.E., HARRIS, A.L., STUART-HARRIS, R.C. & 7 others. (1983). Combination treatment with tamoxifen and aminoglutethimide in advanced breast cancer. *Br. Med. J.*, **286**, 1615.

STOLL, B.A. (1981). Breast Cancer: rationale for endocrine therapy. In: *Hormonal Management of Endocrine-Related Cancer*. (Ed. Stoll), Lloyd-Luke, London, p. 77.

VERMEULEN, A. (1976). The hormonal activity of the postmenopausal ovary. *J. Clin. Endocrinol. Metab.*, **42**, 247.

WAKELING, A.E., O'CONNOR, K.M. & NEWBOULT, E. (1983). Comparison of the biological effects of tamoxifen and a new antioestrogen (LY 117018) on the immature rat uterus. *J. Endocrinol*, **99**, 447.

WILLIS, K.J., LONDON, D.R., WARD, H.W.C., BUTT, W.R., LYNCH, S.S. & RUDD, B.T. (1977). Recurrent breast cancer treated with the antioestrogen tamoxifen: correlation between hormonal changes and clinical course. *Br. Med. J.*, **i**, 425.